# Towards Novel Geneless Approaches for Therapeutic Angiogenesis

**DOI:** 10.3389/fphys.2020.616189

**Published:** 2021-01-20

**Authors:** Francesco Moccia, Maria Rosa Antognazza, Francesco Lodola

**Affiliations:** ^1^Department of Biology and Biotechnology “L. Spallanzani”, University of Pavia, Pavia, Italy; ^2^Center for Nano Science and Technology @PoliMi, Istituto Italiano di Tecnologia, Milan, Italy; ^3^Department of Biotechnology and Biosciences, University of Milano-Bicocca, Milan, Italy

**Keywords:** cardiovascular disease, therapeutic angiogenesis, endothelial colony forming cells, intracellular Ca^2+^ signaling, transient receptor potential vanilloid 1, cell fate, optical stimulation, conjugated polymers

## Abstract

Cardiovascular diseases are the leading cause of mortality worldwide. Such a widespread diffusion makes the conditions affecting the heart and blood vessels a primary medical and economic burden. It, therefore, becomes mandatory to identify effective treatments that can alleviate this global problem. Among the different solutions brought to the attention of the medical-scientific community, therapeutic angiogenesis is one of the most promising. However, this approach, which aims to treat cardiovascular diseases by generating new blood vessels in ischemic tissues, has so far led to inadequate results due to several issues. In this perspective, we will discuss cutting-edge approaches and future perspectives to alleviate the potentially lethal impact of cardiovascular diseases. We will focus on the consolidated role of resident endothelial progenitor cells, particularly endothelial colony forming cells, as suitable candidates for cell-based therapy demonstrating the importance of targeting intracellular Ca^2+^ signaling to boost their regenerative outcome. Moreover, we will elucidate the advantages of physical stimuli over traditional approaches. In particular, we will critically discuss recent results obtained by using optical stimulation, as a novel strategy to drive endothelial colony forming cells fate and its potential in the treatment of cardiovascular diseases.

## Introduction

The vascular network is indispensable for all organisms to distribute oxygen (O_2_) and nutrients to the tissues and to remove carbon dioxide and other metabolic waste products (Heinke et al., 2012). Additionally, the circulatory system serves to maintain homeostasis by stabilizing body temperature and avoiding pH unbalance, to facilitate inter-organ humoral communication, and finally, to guide immune cells towards sites of inflammation or infection (Heinke et al., 2012; Udan et al., 2013). Insufficient vascularization or impairment of regional blood flow due to local vessel obstruction results in ischemia, thereby promoting coronary artery disease, acute myocardial infarction, peripheral artery disease, stroke, pre-eclampsia, and obesity- or neurodegenerative associated disorders (Draoui et al., 2017; Potente and Mäkinen, 2017). Cardiovascular disease (CVD) induced by disruption of the vascular network in heart, limbs and brain is, therefore, regarded as a global medical and economic issue with high prevalence and mortality rates (Benjamin et al., 2019). The World Health Organization and Global Burden Disease have listed CVD as the first cause of death worldwide (Mensah et al., 2019). Therapeutic angiogenesis (TA) represents a promising strategy that aims at reconstructing the damaged vascular network by stimulating the regrowth of the endothelial cell layer that lines the inner lumen of blood vessels and plays a crucial role in adjusting blood supply according to local energy demand (Qadura et al., 2018; Prasad et al., 2020). Endothelial colony forming cells (ECFCs), which represent the only known truly endothelial precursor (Medina et al., 2017), are mobilized in peripheral circulation to maintain endothelial homeostasis throughout postnatal life and to rescue local blood flow upon an ischemic insult (D’Alessio et al., 2015; Tasev et al., 2016; O’Neill et al., 2018). A wealth of *in vitro* and *in vivo* studies has been recently carried out to design an effective strategy to stimulate endogenous ECFCs’ regenerative potential for therapeutic purposes, thereby alleviating the life-threatening impact of CVD (Tasev et al., 2016; Moccia et al., 2018a; O’Neill et al., 2018; Paschalaki and Randi, 2018).

In this perspective, we will briefly describe how endothelial precursors generate the primitive vascular plexus and can, therefore, be exploited for TA. Then, we will explain the rationale for targeting the Ca^2+^ handling machinery, which delivers a crucial pro-angiogenic signaling input. Finally, we will review recent approaches, based on the use of physical stimuli in place of chemical cues. Specifically, we will report on the use of visible light pulses to stimulate ECFCs’ proliferation and bidimensional tube formation. Optical modulation could provide an effective strategy to rescue ECFCs’ vasoreparative potential in patients affected by CVD and to circumvent the main hurdles associated to autologous stem cell therapy.

## The Role of Ecfcs in Vascular Development and Homeostasis: Origin, Characterization, and Suitability of Therapeutic Angiogenesis

The circulatory system is the first functional organ to develop (already during gastrulation) with the purpose to supply growing tissues with O_2_ and nutrients and thereby sustain organism growth (Udan et al., 2013; Potente and Mäkinen, 2017). Embryonic blood vessels arise from endothelial progenitor cells (EPCs), also known as angioblasts, which differentiate from multipotent mesodermal precursors. EPCs coalesce and assembly into a primitive capillary plexus, according to a process known as vasculogenesis. This is followed by further expansion of the vascular network *via* angiogenesis, which may occur through either sprouting or splitting of pre-existing vessels (Udan et al., 2013; Potente and Mäkinen, 2017). The endothelial monolayer retains a state of proliferative quiescence for years, but it may undergo sprouting angiogenesis to meet local metabolic demand under hypoxia, i.e., during skeletal muscle exercise, or in the cycling ovary and in the placenta during pregnancy (Potente and Mäkinen, 2017). Furthermore, EPCs may be released on demand by cytokines released from hypoxic/injured tissues to support local angiogenesis and rescue local blood flow (Moccia et al., 2012; O’Neill et al., 2018). Since the landmark discovery of a population of endothelial precursors circulating in peripheral blood (Asahara et al., 1997), multiple EPC subtypes were isolated, characterized and probed for their therapeutic potential (Asahara et al., 2011; Keighron et al., 2018). Nevertheless, ECFCs were recently presented as the most suitable cellular substrate for regenerative therapy of CVD (Moccia et al., 2015; Tasev et al., 2016; Medina et al., 2017; Paschalaki and Randi, 2018; O’Leary et al., 2019). Unlike other myeloid EPC subtypes, which stimulate neovessel growth in a paracrine manner, ECFCs display the following properties: (1) they are truly endothelial progenitors, able to assembly into capillary-networks *in vitro* and to form patent vessels *in vivo*; (2) display high clonogenic potential and may be replated into secondary and tertiary colonies; (3) rescue injured vascular networks by physically engrafting within neovessels and by releasing pro-angiogenic signals; (4) interact with perimural cells, which ensures neovessel stability; and (5) are more amenable for pharmacological and genetic manipulation aiming at improving their vasoreparative phenotype (Moccia et al., 2015, 2018a,b; Tasev et al., 2016; Medina et al., 2017; Paschalaki and Randi, 2018; O’Leary et al., 2019).

## Intracellular Ca^2+^ Signaling Drives ECFCs’ Angiogenic Activity

**Figure 1:**
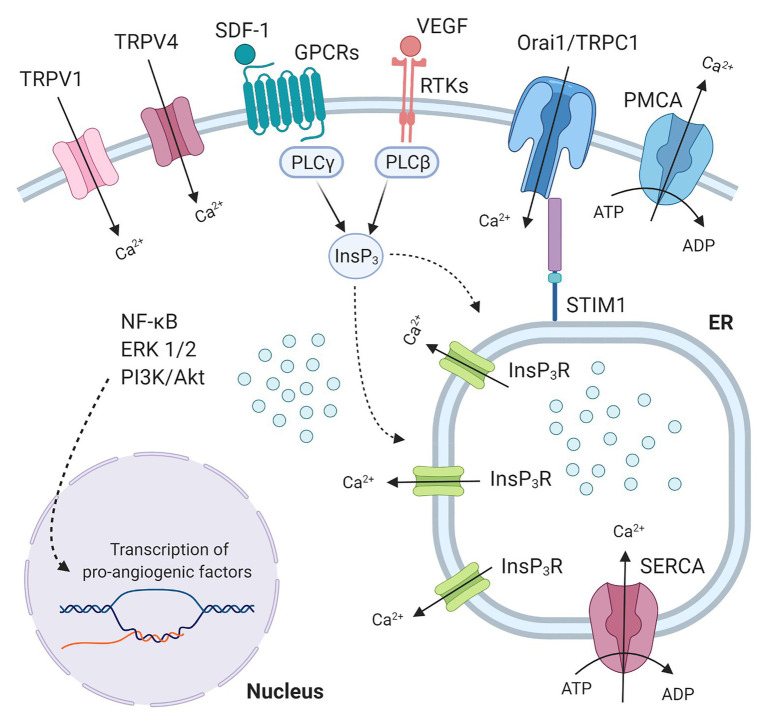
Endothelial colony forming cells (ECFCs) Ca^2+^ machinery and pro-angiogenic Ca^2+^ signals. Growth factors (like VEGF or IGF2) and chemokines (like SDF-1) bind to Receptor tyrosine kinases (RTKs) and G protein-coupled receptors (GPCRs) respectively, thus activating specific PLC isoforms, which in turn leads to production of inositol 1,4,5-trisphosphate (InsP_3_). InsP_3_ binds to InsP_3_ receptors (InsP_3_R) bearing the release of Ca^2+^ from the endoplasmic reticulum (ER) pool. The Ca^2+^ store depletion, detected by Ca^2+^ sensor Stromal interaction molecule 1 (STIM1), is the signal for store-operated calcium entry (SOCE) activation. SOCE, the major Ca^2+^ entry pathway in ECFCs, is mediated by the interaction among STIM1 and the proteins Orai1 and Transient receptor potential canonical 1 (TRPC1). These plasma membrane pore channels allow Ca^2+^ entry from the extracellular space that will be subsequently transported from the cytosol within the SR by the Sarco-Endoplasmic Reticulum Calcium ATPase (SERCA), while Plasma membrane Calcium ATPase (PMCA) contributes to clear cytosolic Ca^2+^ levels. Transient receptor potential vanilloid (TRPV) channels (TRPV1 and TRPV4) also represent an alternative pathway for extracellular Ca^2+^ entry in ECFCs. These intracellular Ca^2+^ signals evoke pathways (NF-κB, ERK1/2, PI3K/Akt) that lead to nuclear transcription of pro-angiogenic factors.

A finely tuned spatio-temporal increase in intracellular Ca^2+^ concentration [(Ca^2+^)_i_] in vascular endothelial cells has long been known to stimulate angiogenesis (Fiorio Pla and Munaron, 2014; Moccia et al., 2014, 2019; Negri et al., 2020a). Endothelial Ca^2+^ signals may indeed mediate the pro-angiogenic effect of multiple growth factors, including vascular endothelial growth factor (VEGF; Potenza et al., 2014; Yokota et al., 2015; Savage et al., 2019), and epidermal growth factor (Moccia et al., 2003), inflammatory mediators, such as ATP (Moccia et al., 2001), and pleiotropic hormones, such as erythropoietin (Yu et al., 2017). Likewise, a recent series of reports documented that intracellular Ca^2+^ signals stimulate ECFCs to undergo angiogenesis both *in vitro* (Zuccolo et al., 2016; Lodola et al., 2017a; Wu et al., 2017) and *in vivo* (Zuccolo et al., 2018; Balbi et al., 2019). For instance, VEGF-induced intracellular Ca^2+^ oscillations stimulated ECFC proliferation and tube formation by promoting the nuclear translocation of the Ca^2+^-sensitive transcription factor, nuclear factor-κB (NF-κB; Dragoni et al., 2015b; Lodola et al., 2017a), whereas biphasic Ca^2+^ signals favored stromal derived factor-1α (SDF-1α)-induced ECFC homing to injured tissues by recruiting the extracellular signal-regulated kinase (ERK) and phosphoinositide 3-kinases (PI3K)/Akt (Zuccolo et al., 2018). The Ca^2+^ response to these pro-angiogenic cues was initiated by endogenous Ca^2+^ release from the endoplasmic reticulum (ER) through inositol-1,4,5-trisphosphate (InsP_3_) receptors (InsP_3_Rs), followed by store-operated Ca^2+^ entry (SOCE) activation (Lodola et al., 2017a; Zuccolo et al., 2018; Figure 1). SOCE is activated upon InsP_3_-induced ER Ca^2+^ depletion to refill the ER with Ca^2+^ and is mediated by the interplay among STIM1, Orai1, and Transient Receptor Potential (TRP) Canonical 1 in ECFCs (Lodola et al., 2012; Figure 1). TRP channels provide an alternative pathway for extracellular Ca^2+^ entry in both vascular endothelial cells (Negri et al., 2020a) and ECFCs (Inoue and Xiong, 2009; Hofmann et al., 2014; Dragoni et al., 2015a; Figure 1). Endothelial cells use TRP channels to sense the local microenvironment in which they reside, thereby adapting to subtle changes in the chemical composition of the extracellular milieu and/or in the mechanical forces acting on the vascular wall (Genova et al., 2020; Negri et al., 2020a). For instance, the endothelial TRPV1 is sensitive to an increase in local temperature above 43°C (Negri et al., 2020b) and/or in local hydrogen peroxide (H_2_O_2_; DelloStritto et al., 2016), whereas TRPV4 is sensitive to physical stimuli, such as shear stress (Schierling et al., 2011) and pulsatile stretch (Thodeti et al., 2009), and to arachidonic acid (AA) production (Fiorio Pla et al., 2008). Recent studies suggested that TRP channels may also stimulate ECFCs’ angiogenic activity. For instance, TRPV1-mediated uptake of anandamide stimulates ECFC migration (Hofmann et al., 2014), whereas TRPV4-mediated nitric oxide release promotes the pro-angiogenic effects of AA (Zuccolo et al., 2016). It has, therefore, been suggested that targeting TRP channels could represent an efficient strategy to boost ECFCs’ regenerative potential (Moccia et al., 2015, 2018a). Indeed, TRP channels are physically coupled to specific Ca^2+^-dependent effectors which translate extracellular Ca^2+^ entry through specific pathways into precise biological outputs which differentially affect endothelial cell fate (Smani et al., 2018; Genova et al., 2020; Negri et al., 2020a).

## Current Limitations of Ecfcs for Therapeutic Angiogenesis

ECFCs hold great promise for TA. Conversely, clinical trials clearly showed that cell therapy based upon transplantation of myeloid EPCs fail to induce a remarkable improvement in capillary density and local blood flow in patients affected by CVD (Moccia et al., 2012; Prasad et al., 2020). Indeed, an array of hurdles hampered the enthusiasm towards ECFC introduction in therapy. Firstly, the frequency of circulating ECFCs is rather low, ranging from 0.28 to 15 ECFCs/10^7^ mononuclear cells, which is insufficient to achieve a therapeutically relevant outcome (Moccia et al., 2017, 2018a). Secondly, ECFCs’ angiogenic activity is severely compromised by CVD (Sung et al., 2013; Mauge et al., 2014; Su et al., 2017; Komici et al., 2020) and by cardiovascular risk factors (Shelley et al., 2012; Jarajapu et al., 2014; Mena et al., 2018), which may ultimately lead to ischemia-related disorders. Thirdly, ECFCs’ angiogenic activity could be further reduced once they reach the harsh microenvironment of ischemic tissues. For instance, ECFC proliferation and tube formation are affected in the presence of elevated pro-inflammatory signaling (Mena et al., 2018), oxidative stress (Gremmels et al., 2017), and hypoxia (He et al., 2018; Tasev et al., 2018). As recently reviewed (Faris et al., 2020), the therapeutic use of umbilical cord blood-derived ECFCs, which display a greater pro-angiogenic potential as compared to circulating ECFCs, is currently not feasible for the high cost of their processing and banking and potential immune complications. It has, therefore, been proposed that the therapeutic outcome of ECFCs-based treatment of CVD could be remarkably improved by boosting the specific pro-angiogenic signaling pathways of circulating ECFCs (Tasev et al., 2016; Moccia et al., 2018a,b; Paschalaki and Randi, 2018).

## Strategies to Boost Angiogenesis Based on Physical Stimuli

The evidence that ECFC harvested from CVD patients often present a dysfunctional phenotype with low proliferative potential and reduced vasculogenic and angiogenic capability boosted numerous efforts to improve ECFC therapeutic efficacy (Paschalaki and Randi, 2018). The large majority of these trials relies on a chemical approach, and include epigenetic activation through stimulation of proangiogenic signaling pathways by specific drugs, as well as administration of bioactive compounds (i.e. fucoidan, genistein, globular adiponectin; Tasev et al., 2016). Very recently, acidic preconditioning has been also reported to have positive effects on ECFC adhesion, vascular density and inflammation reduction (Mena et al., 2018). Chemically controlled methods proved to be successful in many cases. Unfortunately, they are mostly considered to be still insufficient to modulate ECFCs’ activity and to promote TA in a fully satisfactory way. In more detail, their critical limitations consist in limited spatial and temporal resolution of administration, as well as lack of reversibility. Thus, the opportunity to employ physical stimuli has been emerging in the latest years as an alternative, innovative tool to control ECFC fate. Several possibilities are being explored in this direction. First, the effects of micropatterning and nano-patterning and, more generally, of mechanical cues, is under intensive investigation. The hypothesis that the direct micropatterning of ECFCs induces morphological elongation, cytoskeletal alignment, and changes in immunogenic and thrombogenic–related gene expression, is being tested. It was recently reported that ECFCs cultured on top of micropatterned polyurethane substrates show sizable alignment to the underlying substrate geometry, accompanied by the alignment of actin fibers and microtubules. However, this did not correspond to significant cellular elongation in the case of ECFCs, nor to sizable changes in the expression of the transcription factor Krüppel-like Factor 2 (KLF-2) or its downstream targets (Hagen and Hinds, 2020). Conversely, in another work, cells patterned on 25 μm-wide lanes, created by alternating collagen-I and a blocking polymer, clearly displayed elongation, and actin alignment. Micropatterning increased their packing densities, without affecting the apoptotic rate, and KLF-2 gene expression was increased in micropatterned relative to non-patterned ECFCs after 50 h. No significant differences were seen in the other genes tested (Hagen and Hinds, 2019). Lower, sub-micrometric scale was also addressed; patterning of ECFCs in this case lead to a decrease in the ECFC area and perimeter, as well as to an increase in their filopodial outgrowth, associated with a modulation of the focal adhesions and overexpression of the ROCK gene (Cui et al., 2018). Overall, however, the number of studies addressing the use of mechanical stimuli on ECFCs is still very limited and does not allow for sketching a complete picture of their effects.

A second possible approach, still in the early stages, is the use of electromagnetic stimulation. It was reported that electrical stimulation, provided by a wearable solar cell, favored the secretion of angiogenic growth factors and EPC migration (Jeong et al., 2017). Moreover, electrical stimulation promoted the formation of capillaries and arterioles in a mouse model of ischemia, while attenuating muscle necrosis and fibrosis and eventually preventing loss of the injured limb. Interestingly, it was also reported that electrical stimulation significantly increases, among other effects, the number of EPCs in the peripheral blood of rats subjected to fluid percussion injury (Zheng et al., 2017). Magnetic field-guided transplantation of silica-coated magnetic iron oxide nanoparticle-labeled EPCs was associated with their enhanced aggregation in the infarcted border zone (Zhang et al., 2019). These initial, promising results are expected to boost the investigation of electromagnetic stimulation in the field of TA, and in more detail the investigation of the effects of a localized electromagnetic field on ECFC activity.

Thirdly, the use of light stimuli may be perfectly suited for TA. In the last decade, the scientific community has exploited the use of light to control the activity of different cell types genetically modified to express light-sensitive ion channels, thus gaining an unprecedented control in terms of selectivity and reversibility (Knollmann, 2010; Deisseroth, 2011). An alternative strategy, that obviates the need of viral gene transfer is based on the use of hybrid interfaces between living cells and organic semiconductors (OS), used as artificial light transducers (Rivnay et al., 2017; Di Maria et al., 2018; Fang et al., 2020; Ohayon and Inal, 2020). OS, and thiophene-based materials in particular, have emerged as promising tools for biological application, thanks to a series of key-enabling characteristics: they are soft materials with a high degree of mechanical conformability; they are highly biocompatible and very well tolerated within *in vivo* conditions; they support both electronic and ionic charge conduction; they are sensitive to visible and near-infrared light; they are easily processed from solution. Among other materials, it has been demonstrated that optical excitation of regioregular Poly (3-hexyl-thiophene), P3HT, reliably and efficiently modulates the activity of living cells, tissues and systems, including non-excitable (Benfenati et al., 2014; Martino et al., 2015) and excitable cells (Ghezzi et al., 2011; Feyen et al., 2016; Lodola et al., 2019b), retinal explants (Ghezzi et al., 2013), as well as invertebrate (Tortiglione et al., 2017) and mammal animal models (Maya-Vetencourt et al., 2017). It has been also reported that illumination of thiophene thin films leads to a functional interplay with cytochrome C protein, opening the path to selective targeting of sub-cellular organelles (Abdel Aziz et al., 2020).

## Optical Control of ECFC Fate Mediated by P3HT

Interestingly, it was demonstrated that optical excitation of P3HT leads to sizable modulation of TRPV1 channels, in TRPV1 Stable Cell Line-HEK-293 (Lodola et al., 2017b). Moreover, we unambiguously proved that optical excitation of thiophene-based materials leads to non-toxic activation of photoelectrochemical phenomena (Tullii et al., 2017; Abdel Aziz et al., 2020), i.e., reactive oxygen species (ROS) generation and subsequent modulation of Ca^2+^ dynamics (Bossio et al., 2018; Moros et al., 2018). Indeed, reduction of the oxygen present in the extracellular medium in consequence to the polymer photoexcitation leads to the formation of superoxide (O_2_^−^), intermediate ROS and ends up with spatially and temporally controlled generation of H_2_O_2_, which, in turn, can permeate the plasma membrane, thereby causing an increase in the cytosolic H_2_O_2_ levels, which can activate TRPV1 and induced extracellular Ca^2+^ entry (DelloStritto et al., 2016; Lodola et al., 2019a). A local reduction in extracellular pH because of polymer photoexcitation could also gate TRPV1 (Negri et al., 2020b), but its role in P3HT-mediated TRPV1 activation remains to be investigated.

**Figure 2:**
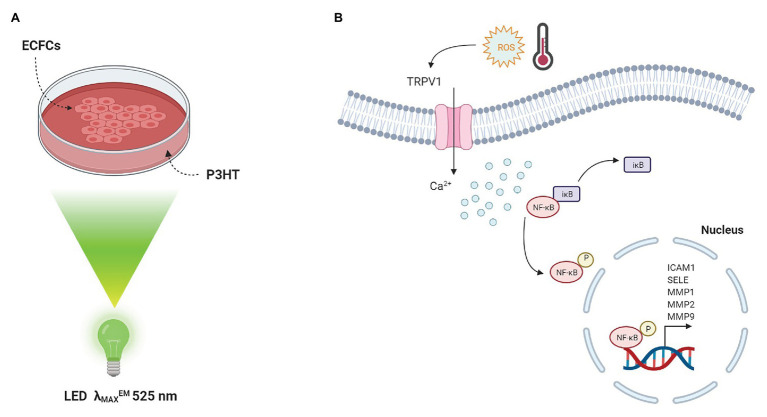
Conjugated polymers optically drive the fate of Endothelial Colony Forming Cells. **(A)** Sketch of the polymer device used for cell optical activation. ECFCs are cultured on top of P3HT thin films, deposited on glass substrates. Optical excitation is provided by a green LED (λ_MAX_^EM^ 525 nm). **(B)** Photo-thermal and photo-electrochemical reactions occur at the interface between material and ECFC membrane. The latter is the predominant mechanism triggering TRPV1 activation. A subsequent increase in [Ca^2+^]_i_ results in the degradation of IκB, the inhibitory sub-unit of the transcriptional factor NF-κB. As a consequence, the p65 NF-κB subunit is released from IκB inhibition and translocates into the nucleus leading to a robust up-regulation of angiogenic genes, which are under NF-κB-dependent transcriptional control.

This experimental evidence prompted us to investigate whether a similar optically-triggered approach could have a beneficial effect on the modulation of ECFC’s angiogenic activity. To this purpose, circulating ECFCs were seeded on top of P3HT and subjected to light stimulation in the green visible region (Figure 2A). We observed that P3HT excitation leads to spatiotemporally resolved modulation of the Ca^2+^ permeable TRPV1 channel, as well as increased ECFC proliferation and tubulogenesis (Lodola et al., 2019a). The interplay among these experimental evidences was clarified by means of a detailed pharmacological analysis: TRPV1 inhibition and manipulation of intracellular free Ca^2+^ levels by selective drugs impaired the pro-angiogenic effect of P3HT excitation thus highlighting the pivotal role of TRPV1-mediated Ca^2+^ influx in ECFC proliferation and tube formation. Moreover, we experimentally identified the phototransduction effect as due to a temporally and spatially localized activation of photoelectrochemical reactions at the interface between the conjugated polymer surface and the cell membrane. Finally, we depicted the molecular scenario observing that polymer photoexcitation led to a significant nuclear translocation of the Ca^2+^-sensitive transcription factor NF-kB and subsequent up-regulated the mRNA levels of specific pro-angiogenic genes (Figure 2B). Overall, these results start paving the way towards the use of conjugated polymers as reliable and efficient functional materials for precise and reversible optically-driven modulation of ECFC physiological activity.

## Conclusion

In this perspective, we have summarized the most recent outcomes in the field of TA. ECFCs are emerging as suitable candidates for cell-based therapy, but to achieve clinically relevant results it is pivotal to ameliorate current treatment limitations (i.e., insufficient circulating ECFCs frequency, impaired angiogenic activity in CVD, low engraftment, survival and integration within the inhospitable environment of damaged myocardium). The use of physical stimuli, a still less beaten path that should ideally receive increasing attention in the forthcoming years, may allow to overcome these drawbacks. The development of novel biohybrid interfaces between ECFC and materials endowed with electrical, photoacoustic, piezoelectric, magnetic, and/or optical properties may reveal a successful route for selective stimulation of pro-angiogenic signaling pathways. The portfolio of different possibilities is still fully open and among them, the use of optical stimuli represents a minimally invasive strategy, able to trigger the desired biophysical pathways with unprecedented selectivity and spatial resolution. In particular, the promising results shown by ECFC optical stimulation using light-sensitive conjugated polymers (Lodola et al., 2019a) may be further exploited in multiple directions. Optical stimulation could be harnessed to stimulate also capillary endothelial cells nearby the injury site, thereby promoting local angiogenesis. Besides circulating ECFCs, TRPV1 is largely expressed and drives proliferation and tube formation in vascular endothelial cells (Negri et al., 2020a,b). Light active materials can be easily patterned with micro- and sub-micrometer resolution, and processed in three-dimensional architectures (Tullii et al., 2020). Another possible action consists in the development of optically active beads, eventually functionalized with specific moieties, for the selective targeting of ECFCs. Polymer nanoparticles can be easily internalized within cells, can target subcellular organelles, show excellent photocatalytic properties, and are able to modulate intracellular Ca^2+^ dynamics and display optimal *in vivo* biocompatibility properties (Bossio et al., 2018; Maya-Vetencourt et al., 2020). Thus, they may serve as sub-micrometer active sites for local triggering of ECFC pathways relevant for TA. Moreover, conjugated polymers are prone to chemical functionalization with specific drugs, opening the possibility to couple optical excitation with on-demand pharmacological treatment. Many crucial issues should be carefully addressed in detail before any preclinical test can be envisaged: (i) understand the complex interplay among materials, physical stimuli and ECFCs biophysical pathways, e.g., the investigation of additional ROS-sensitive pro-angiogenic channels, such as TRP Melastatin 2 (Mittal et al., 2015); (ii) critically evaluate the dose-response efficiency and reliability of the different approaches and stimulation devices; (iii) assess any possible biocompatibility issue and adverse side effects; (iv) develop suitable tools for implantation and *in vivo* chronic use (i.e., engineering of proper waveguides as well as implementation of microscopic, minimally invasive light sources already optimized for optogenetics). Experimental studies in this direction, though highly promising, are currently at a very embryonal stage, and in our opinion would deserve supra-disciplinary efforts from the bioengineering, materials science, and physics communities. We believe the effort will be worth taking and will pay off in time.

## Data Availability Statement

The original contributions presented in the study are included in the article/supplementary material, further inquiries can be directed to the corresponding author.

## Author Contributions

All authors listed have made a substantial, direct and intellectual contribution to the work and approved it for publication.

### Conflict of Interest

The authors declare that the research was conducted in the absence of any commercial or financial relationships that could be construed as a potential conflict of interest.
